# Medical Opinion and Movement

**Published:** 1908-07-18

**Authors:** 


### Medical Opinion and Movement.

Staehelin is not sure that the problem has been solved as to whether a high temperature is harmful or the reverse in such diseases as exhibit this as a constant symptom. Bacteriology has shown that, as a rule, those animals resist best whose temperature rises after inoculation experimentally with organisms. The rule is, however, not absolute. It is not justifiable to conclude from this that all antipyretics are harmful or useless. They are indicated when the patient is suffering from insomnia, delirium, coma, etc. One must be guided in these cases not by the degree of fever, but by the effects that the high temperature produces on the patient. The question as to whether the treatment employed should be that of drugs or that of physical apparatus, such as cold packs, cold baths, iced water, Leiter's coils, etc., can only be settled by the nature of the case. If high temperature is causing urgent symptoms, drugs are too slow to be of use. In typhoid fever hydrotherapy is indicated as being the most rapid method. Moreover, the effects are excellent although the temperature is not lowered, and a bath does good even when the water is not kept strictly at a temperature of from 70° to 85° F. Elimination is promoted by this treatment and sleep encouraged, and the circulatory and nervous systems are improved in tone. The usual antipyretic drugs act by lowering the temperature and improving metabolism. The author is of opinion that all exaggeration should be avoided in combating high temperature, and that each case should be judged on its own merits when the choice of treatment has to be determined upon.

---

Cholesterine, according to M. H. Iscovesco, protects the red blood-corpuscles of man from the harmful action of the serum and hæmolytic agents. This protective action is due to two factors: (1) Cholesterine diminishes the hæmolytic power of serum; (2) it augments the resistance of the erythrocytes. The author, taking up his position upon the result of various experiments, has employed cholesterine as a therapeutic agent in several affections. He administers daily doses of one and a half to two grammes. His twenty cases included purpura rheumatica, chlorosis, tuberculosis, and infantile lymphatism with adenopathies. All patients so treated were either cured or their condition markedly improved.

---

The routine use of the infant's binder has lately been condemned in one or two quarters, but there is evidence to show that in whooping-cough the judicious application of an abdominal binder is often followed by improvement in the child's condition. This measure was discussed at a recent meeting of the Philadelphia Pediatric Society, and various physicians testified to its value in diminishing the number of paroxysms and reducing the liability to vomiting. Dr. P. B. Cassidy reported 12 cases of pertussis in which the abdominal support afforded by a binder was productive of benefit. He recommends, according to the report in the *British Journal of Children's Diseases*, that stockingette should be used, set in as two side strips in Canton flannel, and that the binder thus constructed should lace up at the back. Dr. Davisson, in the course of the discussion, drew attention to the attitude of the community towards whooping-cough. Public opinion in America appears to be as indifferent to the gravity and infectiousness of this disease as is the case in Great Britain. Dr. Davisson demands the establishment of special hospitals for pertussis, and suitable legislation to prevent its unnecessary dissemination.

---

The psychoses of influenza form the subject of a paper by Dods Brown in the *Scottish Medical and Surgical Journal*. Although influenza is the cause of no special form of psychosis, it appears to be the most important febrile disease in the causation of mental disorder. It is now recognised that cases of pre-existing mental disease are increased in severity by the supervention of influenza. Dr. Dods Brown maintains that post-influenzal exhaustion, although an important factor, is not the only factor in the prejudicial effect of influenza upon the brain. The influenza bacillus or toxin seems to be the exciting agent. But Pfeiffer's bacillus has not yet been found in the cerebro-spinal fluid of patients exhibiting ordinary post-influenzal psychoses, although it has been found in cases complicated by meningitis. Melancholia with pronounced suicidal tendency is the commonest form of insanity which is associated with influenza; but almost any kind of psychosis may be seen. The maniacal type of mental disorder is commonest in women and young children, and comes on earlier than the melancholic variety. In 60 per cent. of cases of post-influenzal insanity Dr. Dods Brown obtained a definite history of predisposition, either by heredity or by a previous attack. He recommends that patients suffering from influenza should be isolated and cared for as infectious cases, as far as possible. It is of importance that they should be thoroughly treated, especially when they are known to have an unstable nervous system. During convalescence much can be done to avert a mental breakdown by change of air and stimulating treatment.

---

Chronic appendicitis is now a well-recognised pathological entity, but it is by no means easy of diagnosis in individual cases. It may give rise to the most diverse and diffuse symptoms, symptoms which are often reflex in origin and but distantly suggest the underlying cause. Indefinite abdominal pains of a more or less spasmodic nature and not localised to the appendicular region, muco-membranous colitis, and other gastro-intestinal troubles may result from a chronic inflammation of the appendix without localising symptoms. Dr. Richelot has recently contributed an instructive paper on the subject, with the reports of several cases illustrating the difficulties in the diagnosis of this condition and the way in which such indefinite abdominal or gastro-intestinal symptoms may disappear on removal of the appendix. In each case the patient was a sufferer for a period of some years before definite localising symptoms set in, enabling the surgeon to place his finger upon the seat of the mischief. It would be unjustifiable to presuppose a faulty appendix in all such conditions; but the cases which he reports in the *Journal de Médecine* teach the importance of keeping continually on the watch for some such sign and operating as soon as the evidence is sufficiently clear and definite.

---

The value of preventive injections of antitoxic serum in the prophylaxis of tetanus has been discussed at some length at recent meetings of the Académie de Médecine. According to M. Vaillard, preventive injections were first introduced into veterinary surgery by Nocard, and the results were so satisfactory that the hope was naturally entertained that similar measures might be of some utility for man. An inquiry showed that in the practice of ten veterinary surgeons during the years 1898 to 1906 13,124 animals received preventive injections. These were given either before surgical operations or after serious wounds resulting from accidents, and in no case was tetanus contracted. On the other hand, the application of preventive injections to man does not appear to have influenced the mortality from tetanus. In Paris mortality statistics show an actual increase in deaths from this disease in recent years, since the practice of preventive injections has become generally adopted in the hospitals. Moreover, numerous cases have been reported in which the disease has developed in spite of injections. In the majority of these cases the disease appeared some time after the injections, and it is now a recognised fact that the serum does not confer immunity for a longer time than about a week. In order, therefore, to prevent the disease after possible infection, the injections should be repeated for several weeks. A further weak point in the treatment lies in the fact that the serum is only antitoxic and not bactericidal, so that the spores can continue to germinate in the body, and as soon as the immunity conferred on the individual by the serum has disappeared the disease develops. In this way the disease may appear 87 days after infection, unless the serum injections have been continued during that interval.

---

The operation of cardiolysis, originally conceived by Professor Brauer in 1903 and successfully carried out for him in three cases by Professor Petersen and Dr. Simon, is undoubtedly a new triumph for modern surgery in its invasion of the physician's sphere. The operation consists in removal of some part of the ribs and costal cartilages of the precordial area, in order to free the heart from the restraint of its bony encasement. In cases of cor bovinum resulting from pericardial and pleuro-pericardial adhesions, it is suggested that the increased work of the heart and resulting cardiac distress are in part due to the impact of the enlarged heart against the hard costal wall. In accordance with this view Professor Brauer devised the operation of cardiolysis, and in the few cases in which it has been carried out the results have been, according to reports, highly gratifying. In addition to the three cases mentioned above, the operation has also been performed by Von Beck, König, Lindner, Küttner, and Danielsen. Quite recently Dr. Urban reports a further success. The patient was a young man aged twenty-two, who had suffered from rheumatic fever six years previously. On admission to the hospital he had all the signs and symptoms of a much-dilated and failing heart. Dyspnœa, cyanosis, venous pulsation, general œdema, enlarged liver and spleen, and a systolic bruit at the apex, together with a much-extended area of precordial dulness. As the condition did not yield to ordinary medical treatment the operation of cardiolysis was performed and portions of the fourth, fifth, and sixth ribs and costal cartilages were removed. As a result all the general signs of cardiac failure disappeared, and when the patient left the hospital a month later he was able to get about without distress and even mount the stairs rapidly and without fatigue. Percussion and radioscopy also showed a remarkable diminution in the size of the heart.

---

Still more recently a similar case is reported in this country by Dr. Alexander Morison, of the Great Northern Central Hospital. The patient was also a young man aged nineteen, who had suffered from aortic regurgitation for some years. In addition to the usual bruits at the base and a presystolic murmur at the apex, the heart showed considerable increase in size and the most urgent symptoms were cardiac pain and severe anginal attacks of cardiac distress. As these did not yield to treatment and incapacitated the patient for work, Brauer's operation was decided upon and it was carried out by Mr. Stabb. A precordial flap of the superficial tissues was raised from the ribs and considerable portions of the fifth and sixth ribs removed. The patient made a good recovery and has obtained great relief in all respects from the operation, and especially in regard to the previous anginal attacks. In discussing the technique of the operation Dr. Urban remarks that some surgeons resect both the anterior and posterior costal periosteum, as well as part of the sternum and even the intercostal muscles. He follows König, however, in recommending that the deep periosteum should not be touched, except when there are thick adhesions between the pericardium and ribs. By operations on the cadaver he finds that it is impossible to resect the deep periosteum without injury to the pleura. It is interesting to note in this connection that Mr. Stabb did remove the periosteum, and in so doing punctured the pleura. Dr. Morison suggests that thoracostomy is a more suitable term than cardiolysis, seeing that the operation does not relieve the heart of its adhesions, but frees it from impact against the bony thoracic wall. The operation is a highly interesting one, and the results so far are singularly uniform in the marked relief conferred upon the patients.

THE routine microscopical examination of removed organs has brought to light within the past decade many unsuspected cases of malignant disease, as well as proved the innocency of many gross tumours clinically regarded as malignant. In the current issue of the *Annals of Surgery* several papers are devoted to these out-of-the-way instances of malignancy. Dr. R. H. Harte considers primary carcinoma and sarcoma of the appendix, of which he has collected nine hitherto unpublished cases, which bring the total so far recorded to ninety-eight. He concludes that primary carcinoma is present in from 0.3 to 1.0 per cent. of all cases operated on for chronic appendicitis; that it is a condition of comparatively early life (10 to 40 years) which shows little tendency to metastasis; that it causes symptoms only when an inflammatory process is also present, and is probably originated by such inflammation. Dr. Shepherd reports a case of primary melanotic sarcoma of the common bile duct: and Dr. McMurtry reports two cases of primary carcinoma of the female urethra; the latter says that the entire list of this lesion which he has been able to collect totals twenty-seven cases, and he lays stress on the difficulty of diagnosis between carcinoma and the common benign urethral caruncle. There is also difficulty in excluding with certainty syphilitic lesions in the same situation. The treatment, it is hardly necessary to add, is complete excision whenever the diagnosis is made sufficiently early to admit of it.

---

PROFESSOR CRILE, whose work on shock and collapse is so well known, publishes some helpful suggestions on the surgical treatment of acute Graves' disease. It is found that the psychical excitement of an impending operation not uncommonly sets up hyperthyroidism, which may prove fatal soon after the operation or even before it, and in any case leads to difficulty during the induction of anæsthesia. The temperature in these cases is frequently above 107° Fahr. and sometimes reaches 110°. It is recommended that such patients should be asked to consent to operation if medical measures fail, and that, while remedies are being tried, an anæsthetist should every morning administer inhalations of some pleasant volatile oil on an ether mask. If drug treatment fails, the patient is given a large dose of bromide on the evening before operation and an injection of morphia the following morning; he is told nothing about the operation. The same anæsthetist then begins the inhalation treatment, tells the patient it will be a little stronger this time, and gradually substitutes ether for the volatile oil. The patient is then taken to the operating room and the greater part of each lobe removed after tying the blood vessels. It is claimed that the results of thus "stealing" the gland, as Professor Crile calls it, are perfectly satisfactory even in the most acute cases. The only weak point seems to be that any patient whose previous experience included an operation would appreciate the meaning of the necessary preparation of the intestinal tract and of the skin of the neck.

SINCE the appearance of the works of Oertel it has become almost a common practice to restrict the ingestion of liquids by patients suffering from nephritis, in order to facilitate the activity of the heart. Strauss, in the *Berlin Klin. Woch.*, questions the wisdom of this routine form of treatment, and brings forward cases in which patients take far greater quantities of liquid without showing the least trace of any cardiac fatigue. As an example, he gives the case of a patient suffering from diabetes insipidus, who daily imbibes from 20 to 30 pints of fluid without any effect upon his heart. The author is willing to admit that, in cases of dropsy of cardiac origin, treatment by the suppression, as far as possible, of liquids is justifiable. On the other hand, he holds that in renal dropsy no such treatment is admissible. He declares it proved that the ingestion of liquids contributes, in nephritis, to the important function of ridding the organism of toxic substances. Any excess above the normal intake of fluid is a good defence against the onset of uræmia. Cardiac hypertrophy and polyuria in nephritis constitute a compensatory mechanism against the uræmic tendency. Of course, it is obvious that if the patient be suffering from cardiac dropsy in addition to nephritis the suppression of any excess of liquid from the diet is imperative; in such case small quantities of liquid taken at frequent intervals will suffice. As a general rule, however, it is better to direct one's efforts towards the suppression of uræmia, against which, when it is present, we have few weapons, than with too great energy to combat dropsies which serve to dilute the toxins retained in the organism.

---

FOR operative interference to be of real benefit in carcinoma of the stomach it must be instituted quite at the commencement of the disease. Unfortunately, early diagnosis of the condition is extremely difficult, and in most cases can only be arrived at after a lengthy series of observations involving complicated clinical examinations. All this means much loss of valuable time from the patient's point of view, as well as diminution of the chances of a successful operation if the diagnosis be positive. These considerations have led Prezewalski, of Kharkov, to draw attention to a peculiarity of the intercostal spaces which he discovered in those suffering from carcinoma ventriculi. He was able to persuade himself that whereas in the later stages of the disease all the intercostal spaces on both sides are distinctly retracted, in the early stages of the disease retraction is only to be seen in the central intercostal spaces, and then only on the right side. He attributes this phenomenon to a reflex retraction brought about through the agency of the right vagus nerve, which supplies that portion of the stomach-wall most commonly attacked by cancer. In the case of carcinoma of the œsophagus, the author has never observed a retraction localised entirely to the right intercostal spaces; in such cases the retraction is only found on the left side when the disease is in its early stages, but it may become bilateral in the later stages. The retraction of the intercostal spaces must be sought for by digital palpation, especially in the axillary line.

